# Interplay between Comprehensive Inflammation Indices and Redox Biomarkers in Testicular Germ-Cell Tumors

**DOI:** 10.3390/jpm12050833

**Published:** 2022-05-20

**Authors:** Uros Bumbasirevic, Nebojsa Bojanic, Tatjana Simic, Bogomir Milojevic, Marko Zivkovic, Tijana Kosanovic, Boris Kajmakovic, Aleksandar Janicic, Otas Durutovic, Milan Radovanovic, Veljko Santric, Milica Zekovic, Vesna Coric

**Affiliations:** 1 Clinic of Urology, University Clinical Center of Serbia, 11000 Belgrade, Serbia; urosbu@gmail.com (U.B.); bojanicnebojsa@gmail.com (N.B.); em2bogomir@yahoo.com (B.M.); markoziv91@gmail.com (M.Z.); mockay@gmail.com (B.K.); aleksandarmjanicic@gmail.com (A.J.); odurutovic@gmail.com (O.D.); milan_950@hotmail.com (M.R.); veljkosantric@yahoo.com (V.S.); 2 Faculty of Medicine, University of Belgrade, 11000 Belgrade, Serbia; tatjana.simic@med.bg.ac.rs; 3 Institute of Medical and Clinical Biochemistry, Faculty of Medicine, University of Belgrade, 11000 Belgrade, Serbia; 4 Department of Medical Sciences, Serbian Academy of Sciences and Arts, 11000 Belgrade, Serbia; 5 Radiology Department, The University Hospital ‘Dr. Dragisa Misovic-Dedinje’, 11000 Belgrade, Serbia; tijana.kosanovic@dragisamisovic.bg.ac.rs; 6 Centre of Research Excellence in Nutrition and Metabolism, Institute for Medical Research, National Institute of Republic of Serbia, University of Belgrade, 11000 Belgrade, Serbia

**Keywords:** testicular germ-cell tumors, inflammation, systemic inflammatory response, oxidative stress, redox biomarkers

## Abstract

Sustained and dysregulated inflammation, concurrent tumor-induced immune suppression, and oxidative stress are profoundly involved in cancer initiation, presentation, and perpetuation. Within this prospective study, we simultaneously analyzed the preoperative indices of systemic inflammatory response and the representative byproducts of oxidative DNA, protein, and lipid damage with the aim of evaluating their clinical relevance among patients diagnosed with testicular germ-cell tumors (GCT). In the analytical cohort (n = 88, median age 34 years), neutrophil-to-lymphocyte ratio (NLR), derived neutrophil-to-lymphocyte ratio (dNLR), platelet-to-lymphocyte ratio (PLR), lymphocyte-to-monocyte ratio (LMR), systemic immune-inflammation index (SII), systemic inflammation response index (SIRI), and C-reactive protein (CRP) were significantly altered in patients with a higher tumor stage (*p* < 0.05). Highly suggestive correlations were found between NLR, dNLR, and SII and modified nucleoside 8-OHdG. CRP and albumin-to-globulin ratio (AGR) significantly correlated with thiols group level and maximal tumor dimension (*p* < 0.05). Based on receiver operating characteristic (ROC) curve analyses, all the evaluated pre-orchiectomy inflammation markers demonstrated strong performance in predicting metastatic disease; optimal cut-off points were determined for each indicator. Although further large-scale studies are warranted, inflammatory and redox indices may both complement the established tumor markers and standard clinicopathological prognostic variables and contribute to enhanced personalized risk-assessment among testicular GCT patients.

## 1. Introduction

The vast majority of testicular tumors originate from germ cells, and can be broadly classified into two major histotypes: seminoma and nonseminoma [1]. Although considered a rare tumor type accounting for approximately 1% of all cancers affecting the general male population, testicular cancer has a distinctive age-related incidence rate and represents the most commonly diagnosed solid malignancy among men aged 15 to 40 years [2,3].

Given the multilevel implications of chronic inflammation and oxidative stress in cancer, from tumor initiation to metastasis, there is a biologic rationale and growing scientific interest in the predictive and prognostic relevance of inflammatory and redox biomarkers. In addition to acknowledged acute-phase proteins, such as albumin and C-reactive protein (CRP), particular research and clinical attention has been directed toward the cellular elements of the systemic inflammatory process in peripheral venous blood [4,5]. Nevertheless, single hematologic or protein parameters may be influenced by other physiological and pathological confounding factors, thus compromising marker performance and applicability. Hence, the complex biomarkers featuring two or more inflammatory indices, such as neutrophil-to-lymphocyte ratio (NLR), derived neutrophil-to-lymphocyte ratio (dNLR), platelet-to-lymphocyte ratio (PLR), lymphocyte-to-monocyte ratio (LMR), albumin-to-globulin ratio (AGR), systemic immune-inflammation index (SII), and systemic inflammation response index (SIRI), theoretically provide a more reliable, objective, and robust indication of the host immune response, oncologic status, and outcome [6,7,8,9,10]. Numerous studies have confirmed the relationship between these convenient differential blood cell scores and clinicopathological characteristics, tumor invasiveness, recurrence, and survival in a wide array of malignancies, including urological [8,11,12]. However, there is a paucity of information regarding the redox status of testicular cancer patients [13] and the prognostic relevance of redox biomarkers in such populations. Therefore, the aim of the present study was to address the interplay of systemic inflammatory response and oxidative stress among testicular germ-cell tumor (GCT) patients, and to evaluate the clinical relevance of the preoperative biomarkers of these processes.

## 2. Materials and Methods

### 2.1. Subjects

In total, 113 patients with testicular masses were treated at the Urology Clinic, University Clinical Centre of Serbia, between the years 2020 and 2021. In accordance with the previously elaborated enrollment process, predefined eligibility criteria were applied to the cohort [13]. Patients diagnosed with benign scrotal pathology (epidermal cyst, chronic epididymitis, segmental testicular infarction, testicular atrophy, or Leydig cell hyperplasia; n = 12), non-germ-cell testicular tumors (Leydig cell tumor, Sertoli cell tumor, or adenomatoid tumor; n = 7), and non-testicular malignancy (non-Hodgkin lymphoma; n = 1) were excluded from the study. Furthermore, two patients were disqualified due to incomplete data, and three eligible subjects declined to participate. Accordingly, the final analytical group comprised 88 patients (average age 34, range: 19–54 years) with confirmed testicular GCT [14,15]. Sociodemographic and clinical variables were collected by trained medical staff; additional data were procured from patients’ history charts. The Charlson Comorbidity Index (CCI) was calculated upon detailed medical assessment and diagnosis using the standardized scoring algorithm [16]. Clinical staging was established following the standard tumor, node, metastasis (TNM) classification of the International Union Against Cancer (UICC) [17,18]. 

Informed written consent was obtained from all recruited subjects. The study was conducted in accordance with the institutional ethical board standards (23 November 2020, Approval number 717/9, University Clinical Centre of Serbia, Serbia) and the principles of the Declaration of Helsinki.

### 2.2. Biochemical Assessment

Venous blood samples were collected at admission prior to treatment initiation. The plasma level of 8-OHdG (8-hydroxydeoxyguanosine) was measured using an 8-OHdG ELISA Kit (Elabscience, E-EL-0028) and the plasma level of malondialdehyde (MDA) using an MDA ELISA Kit (Elabscience, E-EL-0060), while the concentration of thiol groups was spectrophotometrically determined following to the method reported by Jocelyn [19,20]. Systemic inflammatory biomarkers (NLR, dNLR, PLR, SII, LMR, SIRI, CRP, and AGR) were determined or calculated from routinely obtained laboratory parameters. 

### 2.3. Statistical Analyses

Statistical data were analyzed using IBM SPSS Statistics 22 (SPSS Inc., Chicago, IL, USA). In this paper, numerical data are presented by measures of variability (standard deviation and range), whereas attributive data are presented in absolute and relative numbers. The differences in continuous data with non-normal distribution were assessed using the Mann–Whitney test, while the associations between the levels of redox and systemic inflammatory biomarkers were analyzed using Spearman’s coefficient of linear correlation. Finally, receiver operating characteristic (ROC) curve analysis was performed to assess and visualize the discriminative ability of pre-orchiectomy inflammatory indices for metastatic disease among testicular GCT patients. The optimal cut-off point for each indicator was determined based on the Youden index that optimizes the classifiers differentiating power when equal weight is attributed to sensitivity and specificity levels, representing the true positive and true false rate, respectively. Statistical hypotheses were analyzed at the level of significance of 0.05.

## 3. Results

The descriptive characteristics of enrolled patients with testicular GCT are summarized in Table 1. The median estimated Charlson Comorbidity Index (CCI) for the cohort was zero, with the utmost score of one indicating the absence of a comorbidity burden that could substantially contribute to oxidative stress.

The difference in preoperative inflammatory and redox biomarkers between testicular GCT patients with lower (Stage I) and higher tumor stages (Stages II + III) are presented in Table 2. As indicated, the values of particular complex inflammatory indices, as well as CRP, were significantly altered in patients at a higher stage (*p* < 0.05). No significant difference in the plasma levels of redox biomarkers was observed between these two groups (*p* > 0.05).

**Figure 1:**
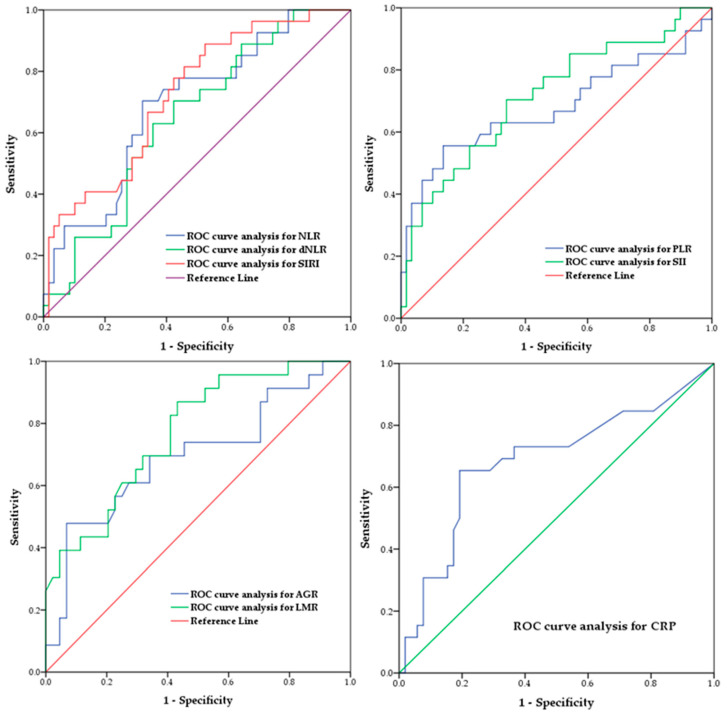
Graphical presentation of receiver operating characteristic (ROC) curve analysis for preoperative inflammatory indices’ performance in predicting metastatic disease among patients diagnosed with testicular GCT.

Based on ROC analyses, all the evaluated pre-orchiectomy inflammation markers demonstrated significance in predicting metastatic disease. The optimal cut-off points, determined for each indicator, are presented in Table 3 along with the corresponding area under the curve (AUC), sensitivity, and specificity levels. ROC curves, as graphical visualizations of the classifiers’ performance, are provided in Figure 1.

The correlation between preoperative inflammatory and redox biomarkers in testicular GCT patients is presented in Table 4. Complex inflammatory indices such as NLR, dNLR, and SII correlated with levels of byproducts of oxidative DNA damage (NLR vs. 8-OHdG rho = 0.210, *p* = 0.067, dNLR vs. 8-OHdG rho = 0.214, *p* = 0.061, and SII vs. 8-OHdG rho = 0.209, *p* = 0.068), whereas CRP and AGR significantly correlated with levels of byproducts of oxidative protein damage (CRP vs. thiol groups rho = −0.234, *p* = 0.042 and AGR vs. thiol groups rho = 0.278, *p* = 0.025). No significant correlation was observed between the levels of oxidative lipid damage (MDA) byproducts and inflammatory biomarkers (*p* > 0.05).

The correlation between preoperative inflammatory and redox biomarkers with tumor maximal dimension was estimated in the analyzed cohort of testicular GCT patients (Table 5). A significant association was observed for CRP and AGR (CRP vs. tumor maximal dimension rho = 0.246, *p* = 0.030 and AGR vs. tumor maximal dimension rho = −0.263, *p* = 0.031).

## 4. Discussion

Sustained and dysregulated inflammation, concurrent tumor-induced immune suppression, and oxidative stress, interconnected via multifarious molecular and cellular mechanisms, are profoundly involved in cancer development, progression, presentation, and prognosis. Although the roles of these processes are well-acknowledged, the literature data regarding the immunocompetence and redox status of testicular cancer patients are scarce. 

Within this study, the preoperative indices of systemic inflammatory response and the most commonly determined byproducts of oxidative DNA, protein, and lipid damage were simultaneously analyzed to evaluate their clinical relevance among patients diagnosed with testicular GCT. Our results showed that NLR, dNLR, PLR, LMR, SII, SIRI, and CRP were significantly altered in patients with higher tumor stages. All the evaluated pre-orchiectomy inflammation markers demonstrated value in predicting metastatic disease. CRP and albumin-to-globulin ratio (AGR) significantly correlated with maximal tumor dimension and thiol group levels. Positive yet insignificant correlations were found between NLR, dNLR, and SII and oxidatively modified nucleoside 8-OHdG.

Cancer-elicited alteration of peripheral blood cell composition is usually presented as an expansion of myeloid subsets, i.e., neutrophils and monocytes, the elevation of platelets, and the decline in lymphocytes [21]. Although underlying mechanisms have not been entirely elucidated, the pro- and anticancer activity of immune cells determines the direction of association between their blood count and prognosis. The advantages of hemocytometric evaluation of single blood elements and derived inflammatory indices are simple availability, routine and reliable laboratory-analytical procedures, minimal financial burden, and an almost complete absence of additional sampling-related risk exposure for patients. Synthesis of multiple markers into complex indices combines synchronized immunohematological changes, presumably providing improved credibility, objectivity, and robustness of assessment. The conceptual goal of the application of these indicators is a simplified presentation of immune function and cancer-associated inflammation, providing potential insight into the complex interplay of these processes and their repercussions for disease development, pathological features, and subsequent clinical outcomes. 

Significantly higher values of comprehensive inflammation indices NLR, dNLR, PLR, SII, and SIRI, and a lower LMR were found in testicular GCT patients at higher tumor stages (Stages II+III) in comparison with subjects presenting at Stage I, reflecting more intensely pronounced proinflammatory activity. There is an emerging scientific interest in the multifaceted role of neutrophils, the most abundant myeloid cells, in cancer regulation. Through various soluble mediators, particularly granulocyte colony-stimulating factor (G-CSF), malignant cells stimulate granulopoiesis in the bone marrow and extramedullary, thereby causing an increase in neutrophils in the circulation and tissues. In addition, cancer modulates their cellular status, degree of maturity, and metabolic and functional characteristics, hence intensifying the role of neutrophils in disease progression [21,22]. Neutrophils facilitate neoplastic transformation by releasing genotoxic substances and causing the accumulation of procarcinogenic genetic instability. Furthermore, these cells promote tumor proliferation via a wide variety of cytokines, chemokines, other soluble factors, and proteinases, and stimulate tumor neovascularization by exerting proangiogenic function [23]. Neutrophils engage in metastatic cascade by potentiating invasive and migratory cancer cell potential, forming cell clusters with advantageous colonization features, and disrupting the extracellular matrix. Finally, neutrophilia modulate immunosuppression by compromising the cytolytic activity of lymphocytes, T cells, and NK cells [24]. Conversely, lymphocytopenia denotes a weakened adaptive immune response to neoplasia and an increased risk of poor prognosis [25]. Experimental and clinical evidence suggests that platelets exhibit protumorigenic activity through a wide spectrum of physical and functional dynamic interactions with cancer and tumor stroma cells. Their distinctive characteristics that facilitate rapid reactivity are their small size and discoid morphology, which maximize the vascular contact area, quantitative abundance in the bloodstream, and diverse biophysical properties including adhesion, aggregation, and efficient migration potential [26]. Platelets may supply cancer cells with pro-oncogenic signals, obstruct the function of effector lymphocytes by modulating the tumor microenvironment, and stimulate cancer cell dissemination [27,28]. 

Systematic reviews and meta-analyses summarized the published literature reports regarding the potential application of NLR in urological cancers, and the pooled evidence suggested that this systemic inflammation index may be considered an effective prognostic predictor, with higher values indicating a worse outcome [12,29,30]. Although additional validation studies are needed, mounting evidence proposes SII as a marker independently related to poor prognosis among patients diagnosed with urologic malignancies [31]. Although limited by the relatively small sample size, two studies reported statistically significantly higher white blood cell counts, NLR, and PLR among testicular cancer patients compared with the cancer-free control groups [32,33]. Furthermore, retrospective cohort studies associated elevated preoperative NLR with adverse pathologic features, advanced tumor staging, higher recurrence, and poorer survival rates in this patient population [34,35,36]. High levels of inflammation markers, namely leukocytes, neutrophils, NLR, and SII, have been credited as valuable oncologic outcome predictors in conjunction with the standard classification system for patients with metastatic germ-cell testicular cancer undergoing first-line chemotherapy [37]. 

In the present study, all the evaluated pre-orchiectomy inflammatory indices demonstrated importance in predicting metastatic disease among patients diagnosed with testicular GCT. As indicated by the ROC analyses, the AUC values for NLR, dNLR, PLR, CRP, and AGR were >0.6, whereas LMR, SIRI, and SII showed superior predictive power with AUC values of 0.730, 0.723, and 0.714, respectively. Although optimal cut-off points were determined for each indicator based on the Youden index, care should be exercised with their potential application. Regardless of the reasonable comparability across studies, there are no standard universally appropriate threshold values for these classifiers. The high degree of heterogeneity of the published data regarding research approaches (study design, observational perspective, statistical processing, transparency, and reproducibility) and clinical indicators (tumor localization and histopathological type, disease stage, therapeutic approach, and analyzed outcomes) hinders the establishment of scientific and professional consensus on this issue. In order to tackle this challenge, there is a need for prospective studies on larger samples with harmonized research designs, adequate control mechanisms for the interfering factors, and uniform end-point variables.

Disruption of the balance between the production of reactive species and antioxidative defense systems may damage cellular macromolecules, precipitating a progressive and cumulative decline in physiological function. In this study, in order to comprehensively address the redox status of testicular GCT patients, thiols group level, 8-OHdG, and MDA were quantified as prototypical biomarkers of protein impairment, DNA modifications, and lipid peroxidation, respectively [38]. Based on correlation analyses, we found that the thiols group level was significantly associated with AGR (directly) and CRP (inversely), reflecting the complex interdependence of oxidative stress and inflammation in testicular cancer. Thiols represent a robust and remarkably versatile protection system against biochemical disturbances induced by oxidative stress. The antioxidant capacity of dynamic thiol–disulfide homeostasis plays a crucial role in the regulation of enzymatic reactions, cellular signal transduction, detoxification, lipid integrity, protein stability, transcription, and apoptosis [39]. Abnormalities in this defense mechanism, manifested as the disruption of thiol–disulfide equilibrium and depletion of thiol compounds, have been confirmed in several malignant conditions such as prostate [40], lung [41], colorectal [42], and gastric cancer [43]. Substantial scientific evidence associates low AGR with an increased risk of wide-spectrum cancer type incidence and mortality [44]. Cumulative exposure to diverse proinflammatory cytokines in cancer-related chronic inflammation causes hyperglobulinemia and suppression of albumin synthesis in the liver [45]. Furthermore, albumin exerts significant extracellular antioxidant properties. This polypeptide chain contains a total of 35 cysteine (Cys) residues, with 34 of them forming 17 intramolecular disulfide bonds. The remaining free one, Cys34, is redox-active and represents the most abundant thiol in plasma [46]. The observed correlations in this study may be attributed to the depletion of thiol groups, decline in the albumin level, and elevation in CRP levels resulting from the synergistical effect of deregulated prolonged inflammation and exposure to an oxidative milieu.

Our findings indicated that the maximal tumor dimension was significantly associated with the protein inflammatory markers, i.e., CRP and AGR, but not with cellular-based indicators. CRP, a hepatically synthesized quintessential, yet nonspecific acute phase reactant, exists in multiple isoforms displaying distinctive functional bioactivities. A recently appreciated notion of the dynamic nature of CRP, referring to its ability to undergo a nonproteolytic conformational transformation, extended the comprehension of its role in cancer-related inflammation amplification and regulation [47]. Pentameric structural conformation (pCRP), the soluble circulating isoform quantifiable in routine diagnostic assessment, promptly and markedly increases as a consequential response to any tissue-damaging process associated with an inflammatory state. In accordance with the presented results, an elevated pCRP level is most seemingly representative of sustained in situ tumor growth, proportionate with the level of subsequent tissue damage [48]. Nevertheless, the complex interaction of CRP with the tumor microenvironment and the neoplasm-surrounding connective tissue and extracellular matrix merit further assessment to illuminate the exact activity, influence, and significance of this inflammatory mediator over the course of disease progression.

Although not reaching the threshold for statistical significance, our data analyses revealed a highly suggestive correlation between hematologic systemic inflammatory indices NLR, dNLR, and SII and modified nucleoside 8-OHdG. These findings indicate that there is an intrinsic interaction between the inflammatory process and DNA damage induced by oxidative stress among testicular GCT patients. Hyperactivated immune system cells secrete inflammatory mediators, which promote genetic impairment and neoplastic transformation, employing various mechanisms including the inhibition of DNA repair pathways and downregulation of scavenging antioxidant activity [49]. In an interdependent manner, oxidative DNA damage may provoke the induction of a signaling cascade, culminating with the activation of the prototypical proinflammatory signaling NF-*κ*B pathway [50]. Guanine is the nucleobase with the lowest redox potential and is therefore most susceptible to oxidation as a result of exposure to reactive oxygen species [51]. Oxidized guanine may form a base pair with adenine via Hoogsteen hydrogen bonding, thus causing a G:C–T:A transversion mutation, which is closely related to tumor development and progression, cell aging, and degenerative pathologies [52,53]. 8-OHdG is intensely implicated in carcinogenesis as the most abundant promutagenic fingerprint of radical attack generating DNA lesions [54,55]. Elevated levels of 8-OHdG were detected among patients with various malignancies such as prostate [56], hepatocellular [57], colorectal [58], oral [59], esophageal [60], gastric [61], and head and neck cancer [62]. Furthermore, a recently conducted quantitative meta-analysis and systematic review showed that highly expressed 8-OHdG in tumor tissues may be considered a valuable prognostic predictor in most solid malignancies [63]. 

Certain limitations of this study should be acknowledged. Although congruent with comparable studies, the single-center design and relatively small sample size may compromise the generalizability of the findings. Furthermore, the investigation of underlying molecular mechanisms, signaling pathway and genetic factors relevant to testicular GCT susceptibility, oxidative stress resilience, and inflammatory response were outside the scope of this research. The potential confounding influence of unidentified clinicopathological issues in the patient cohort could not be excluded. However, the estimated Charlson Comorbidity Index implies the absence of comorbidities that may have biased the observed results. Patients’ tumor tissue and semen samples were not available; hence they were not included in the analyses. Finally, the inherent limitations of inflammatory and redox markers should be addressed. They are susceptible to fluctuations under the influence of other physiological or pathological processes, conditions not related to cancer, previously applied therapeutic procedures, and even specimen harvesting circumstances due to daily intraindividual variability. Preoperative sampling in a controlled clinical setting under standardized conditions and careful patient evaluation was employed to overcome these concerns. Given the novelty of the scientific perspective encompassing surrogate hematological indicators of cancer-associated inflammatory response and biomarkers of oxidative damage, as well as the scarcity of available literature on this issue, the presented findings provide a noteworthy contribution to the field of testicular cancer research.

## 5. Conclusions

Chronic inflammation and oxidative stress are simultaneous, mutually endorsing, and perpetuating processes involved in testicular cancer pathology. Conventional tumor markers support diagnosis, and provide indispensable information for disease staging, risk stratification, monitoring of treatment efficacy, and relapse detection. Nonetheless, inflammation indices derived from complete blood cell count are cost-effective, reliable, and easily accessible without imposing additional laborious efforts. Therefore, these indices may complement established markers and standard clinicopathological prognostic variables and contribute to enhanced personalized risk assessment among testicular GCT patients. However, the potential of redox biomarkers is yet to be clarified. Further larger-scale studies with longer follow-up periods are warranted to corroborate and fully elucidate the complex associations between the systemic inflammatory and redox biomarkers in this patient population.

## Figures and Tables

**Table 1:** The characteristics of patients with testicular GCT.

Parameters	Patients with Testicular GCT, n = 88
Age (years)	34 (19–54) ^1^
Charlsoncomorbidity index, CCI	0 (0–1) ^1^
Body mass index, n (%) ^2^	24.9 (17.6–36.1) ^1^
<30 kg/m^2^	73 (88)
>30 kg/m^2^	10 (12)
Smoking status, n (%) ^2^	
Non-smokers	36 (44)
Smokers	45 (56)
Tumor type, n (%)	
Seminoma	52 (59)
Non-seminoma	36 (41)
Clinical stage, n (%)	
I	61 (69)
II	18 (21)
III	9 (10)

^1^ Median (range); ^2^ Obese patients were characterized as individuals with a body mass index (BMI) above 30 kg/m^2^, whereas smokers were characterized as individuals who were engaged in dailysmoking during any 60-day period, prior to the study onset.

**Table 2:** The difference in preoperative inflammatory and redox biomarkers between testicular GCT patients at lower and higher tumor stages.

Laboratory Parameter	Stage I	Stages II + III	*p*-Value
Neutrophils (n × 10^9^/L)	4.55 (1.80–13.00)	5.20 (2.70–12.22)	0.077
Lymphocytes (n × 10^9^/L)	1.90 (1.10–3.00)	1.60 (0.40–3.32)	0.183
NLR	2.28 (0.72–11.82)	3.28 (1.65–16.00)	0.005 *
dNLR	1.71 (0.58–4.64)	2.18 (1.18–5.77)	0.031 *
Platelets (n × 10^9^/L)	234.00 (102.00–412.00)	260.00 (148.00–562.00)	0.177
PLR	123.68 (67.27–283.64)	163.50 (51.61–640.00)	0.008 *
SII (×10^9^/L)	533.33 (145.71–3687.27)	824.26 (268.39–4096.00)	0.001 *
SIRI (×10^9^/L)	1.06 (0.29–16.36)	1.65 (0.58–6.42)	0.001 *
Monocytes (n × 10^9^/L)	0.48 (0.10–1.30)	0.50 (0.30–1.45)	0.117
LMR	4.20 (0.85–13.00)	3.20 (1.00–5.33)	0.001 *
CRP (mg/L)	0.80 (0.20–301.00)	2.45 (0.20–197.50)	0.008 *
AGR	1.84 (1.23–2.78)	1.62 (0.88–2.35)	0.009 *
8-OHdG (ng/mL)	9.25 (5.07–49.50)	8.51 (4.29–16.48)	0.373
Thiol groups (ng/mL)	9.65 (4.98–218.86)	10.16 (5.93–14.89)	0.914
MDA (ng/mL)	94.01 (41.02–302.85)	98.02 (55.34–372.06)	0.208

NLR—neutrophil-to-lymphocytes ratio; dNLR—derived neutrophil-to-lymphocyte ratio (formula: neutrophil/(WBC-neutrophil)); PLR—platelet-to-lymphocyte ratio; SII—systemic immune-inflammation index (formula: neutrophil × platelet/lymphocyte); SIRI—systemic inflammation response index (formula: neutrophil × monocyte/lymphocyte); LMR—lymphocyte-to-monocyte ratio; CRP—C-reactive protein; AGR—serum albumin/(serum total protein-serum albumin); 8-OHdG—8-hydroxy-2′-deoxyguanosine; MDA—malondialdehyde; * *p* < 0.05.

**Table 3:** Receiver operating characteristics (ROC) curve analysis for pre-orchiectomy inflammatory indices’ performance in predicting metastatic disease among patients diagnosed with testicular GCT.

Inflammatory Index	Cut-Off Point	AUC	*p*-Value	CI	Specificity (%)	Sensitivity (%)
NLR	2.685	0.691	0.05 *	0.573–0.809	67.79	70.37
dNLR	1.775	0.646	0.031 *	0.527–0.765	57.62	70.37
PLR	160.170	0.680	0.008 *	0.539–0.820	86.44	55.55
LMR	4.145	0.730	0.001 *	0.618–0.842	50.84	85.18
SII (×10^9^/L)	683.21	0.714	0.001 *	0.549–0.836	66.10	70.37
SIRI (×10^9^/L)	1.005	0.723	0.001 *	0.612–0.835	47.45	88.88
CRP (mg/L)	2.100	0.684	0.008 *	0.549–0.819	80.76	65.38
AGR	1.530	0.695	0.009 *	0.554–0.838	93.18	44.82

NLR—neutrophil-to-lymphocytes ratio; dNLR—derived neutrophil-to-lymphocyte ratio (formula: neutrophil/(WBC-neutrophil)); PLR—platelet-to-lymphocyte ratio; SII—systemic immune-inflammation index (formula: neutrophil × platelet/lymphocyte); SIRI—systemic inflammation response index (formula: neutrophil × monocyte/lymphocyte); LMR—lymphocyte-to-monocyte ratio; CRP—C-reactive protein; AGR—serum albumin/(serum total protein-serum albumin); AUC—area under the curve; CI—confidence interval; * *p* < 0.05.

**Table 4:** Correlation of preoperative inflammatory and redox biomarkers in testicular GCT patients.

Laboratory Parameter	8-OHdG (ng/mL) Rho/*p*-Value	Thiol Groups (µmol/g) Rho/*p*-Value	MDA (ng/mL) Rho/*p*-Value
Neutrophils (n × 10^9^/L)	0.157/0.172	−0.173/0.115	0.050/0.666
Lymphocytes (n × 10^9^/L)	−0.142/0.217	−0.142/0.197	−0.062/0.595
NLR	0.210/0.067	−0.069/0.531	0.153/0.187
dNLR	0.214/0.061	−0.062/0.575	0.130/0.262
Platelets (n × 10^9^/L)	0.004/0.974	−0.183/0.096	0.088/0.450
PLR	0.132/0.252	0.000/0.997	0.194/0.094
SII (×10^9^/L)	0.209/0.068	−0.118/0.299	0.182/0.116
SIRI (×10^9^/L)	0.144/0.210	−0.129/0.243	0.101/0.385
Monocytes (n × 10^9^/L)	−0.078/0.499	−0.132/0.233	−0.080/0.493
LMR	−0.115/0.320	0.003/0.975	−0.094/0.417
CRP (mg/L)	0.109/0.361	−0.234/0.042 *	−0.049/0.688
AGR	−0.041/0.753	0.278/0.025 *	0.002/0.987

NLR—neutrophil–to-lymphocytes ratio; dNLR—derived neutrophil-to-lymphocyte ratio (formula: neutrophil/(WBC-neutrophil)); PLR—platelet-to-lymphocyte ratio; SII—systemic immune-inflammation index (formula: neutrophil × platelet/lymphocyte); SIRI—systemic inflammation response index (formula: neutrophil × monocyte/lymphocyte); LMR—lymphocyte-to-monocyte ratio; CRP—C reactive protein; AGR—serum albumin/(serum total protein-serum albumin); 8-OHdG—8-hydroxy-2′-deoxyguanosine; MDA—malondialdehyde; * *p* < 0.05.

**Table 5:** Correlation of preoperative inflammatory and redox biomarkers with tumor maximal dimension in testicular GCT patients.

Laboratory Parameter	Tumor Maximal Dimension Rho/*p*-Value
Neutrophils (n × 10^9^/L)	0.175/0.107
Lymphocytes (n × 10^9^/L)	−0.038/0.730
NLR	0.174/0.110
dNLR	0.173/0.110
Platelets (n × 10^9^/L)	0.018/0.869
PLR	−0.004/0.971
SII (×10^9^/L)	0.104/0.339
SIRI (×10^9^/L)	0.120/0.273
Monocytes (n × 10^9^/L)	0.094/0.389
LMR	−0.041/0.711
CRP (mg/L)	0.246/0.030 *
AGR	−0.263/0.031 *
8-OHdG (ng/mL)	0.040/0.729
Thiol groups(ng/mL)	0.064/0.556
MDA (ng/mL)	0.160/0.162

NLR—neutrophil–to-lymphocytes ratio; dNLR—derived neutrophil-to-lymphocyte ratio (formula: neutrophil/(WBC-neutrophil)); PLR—platelet-to-lymphocyte ratio; SII—systemic immune-inflammation index (formula: neutrophil × platelet/lymphocyte); SIRI—systemic inflammation response index (formula: neutrophil × monocyte/lymphocyte); LMR—lymphocyte-to-monocyte ratio; CRP—C reactive protein; AGR—serum albumin/(serum total protein-serum albumin); 8-OHdG—8-hydroxy-2′-deoxyguanosine; MDA—malondialdehyde; * *p* < 0.05.

## Data Availability

The data supporting reported results can be obtained upon request in the form of datasets available at the Clinic of Urology, University Clinical Centre of Serbia, Belgrade, Serbia.

## References

[1] Elzinga-Tinke J.E., Dohle G.R., Looijenga L.H.J. (2015). Etiology and early pathogenesis of malignant testicular germ cell tumors: Towards possibilities for preinvasive diagnosis. Asian J. Androl..

[2] Winter C., Albers P. (2011). Testicular germ cell tumors: Pathogenesis, diagnosis and treatment. Nat. Rev. Endocrinol..

[3] Batool A., Karimi N., Wu X.N., Chen S.R., Liu Y.X. (2019). Testicular germ cell tumor: A comprehensive review. Cell. Mol. Life Sci..

[4] Diakos C.I., Charles K.A., McMillan D.C., Clarke S.J. (2014). Cancer-related inflammation and treatment effectiveness. Lancet Oncol..

[5] Proctor M.J., Morrison D.S., Talwar D., Balmer S.M., Fletcher C.D., O’reilly D.S.J., Foulis A.K., Horgan P.G., Mcmillan D.C. (2011). A comparison of inflammation-based prognostic scores in patients with cancer. A Glasgow Inflammation Outcome Study. Eur. J. Cancer.

[6] Yan Q., Ertao Z., Zhimei Z., Weigang D., Jianjun P., Jianhui C., Chuangqi C. (2020). Systemic immune-inflammation index (SII): A More Promising Inflammation-Based Prognostic Marker for Patients with synchronic colorectal peritoneal carcinomatosis. J. Cancer.

[7] Yamamoto T., Kawada K., Obama K. (2021). Inflammation-related biomarkers for the prediction of prognosis in colorectal cancer patients. Int. J. Mol. Sci..

[8] Zhong J.-H., Huang D.-H., Chen Z.-Y. (2017). Prognostic role of systemic immune-inflammation index in solid tumors: A systematic review and meta-analysis. Oncotarget.

[9] Hu X., Shao Y.X., Yang Z.Q., Dou W.C., Xiong S.C., Li X. (2020). Preoperative systemic immune-inflammation index predicts prognosis of patients with non-metastatic renal cell carcinoma: A propensity score-matched analysis. Cancer Cell Int..

[10] Ying H.Q., Liao Y.C., Sun F., Peng H.X., Cheng X.X. (2021). The role of cancer-elicited inflammatory biomarkers in predicting early recurrence within stage ii–iii colorectal cancer patients after curable resection. J. Inflamm. Res..

[11] Ohno Y. (2019). Role of systemic inflammatory response markers in urological malignancy. Int. J. Urol..

[12] Mjaess G., Chebel R., Karam A., Moussa I., Pretot D., Abi Tayeh G., Sarkis J., Semaan A., Peltier A., Aoun F. (2021). Prognostic role of neutrophil-to-lymphocyte ratio (NLR) in urological tumors: An umbrella review of evidence from systematic reviews and meta-analyses. Acta Oncol..

[13] Bumbasirevic U., Bojanic N., Pljesa-Ercegovac M., Zivkovic M., Djukic T., Zekovic M., Milojevic B., Kajmakovic B., Janicic A., Simic T. (2022). The Polymorphisms of Genes Encoding Catalytic Antioxidant Proteins Modulate the Susceptibility and Progression of Testicular Germ Cell Tumor. Cancers.

[14] Paner G.P., Stadler W.M., Hansel D.E., Montironi R., Lin D.W., Amin M.B. (2018). Updates in the Eighth Edition of the Tumor-Node-Metastasis Staging Classification for Urologic Cancers. Eur. Urol..

[15] Williamson S.R., Delahunt B., Magi-Galluzzi C., Algaba F., Egevad L., Ulbright T.M., Tickoo S.K., Srigley J.R., Epstein J.I., Berney D.M. (2017). The World Health Organization 2016 classification of testicular germ cell tumours: A review and update from the International Society of Urological Pathology Testis Consultation Panel. Histopathology.

[16] Charlson M.E., Pompei P., Ales K.L., MacKenzie C.R. (1987). A new method of classifying prognostic comorbidity in longitudinal studies: Development and validation. J. Chronic Dis..

[17] Brierley J.D., Gospodarowicz M.K., Wittekind C. (2017). TNM Classification of Malignant Tumours.

[18] Laguna M., Albers P., Algaba F., Bokemeyer C., Boormans J., Fischer S., Fizazi K., Gremmels H., Leão R., Nicol D. EAU Guidelines on Testicular Cancer. Proceedings of the 36th Annual EAU Congress.

[19] Jocelyn P.C. (1987). Spectrophotometric assay of thiols. Methods in Enzymology.

[20] Walmsley T.A., Abernethy M.H., Fitzgerald H.P. (1987). Effect of daylight on the reaction of thiols with Ellman’s reagent, 5,5′-dithiobis(2-nitrobenzoic acid). Clin. Chem..

[21] Sionov R.V., Fridlender Z.G., Granot Z. (2015). The Multifaceted Roles Neutrophils Play in the Tumor Microenvironment. Cancer Microenviron..

[22] Bao Y., Liu Z., Guo M., Li B., Sun X., Wang L. (2018). Extramedullary hematopoiesis secondary to malignant solid tumors: A case report and literature review. Cancer Manag. Res..

[23] Zhang X., Zhang W., Yuan X., Fu M., Qian H., Xu W. (2016). Neutrophils in cancer development and progression: Roles, mechanisms, and implications (Review). Int. J. Oncol..

[24] Hedrick C.C., Malanchi I. (2021). Neutrophils in cancer: Heterogeneous and multifaceted. Nat. Rev. Immunol..

[25] Schepisi G., Santoni M., Massari F., Gurioli G., Salvi S., Conteduca V., Montironi R., De Giorgi U. (2016). Urothelial Cancer: Inflammatory Mediators and Implications for Immunotherapy. BioDrugs.

[26] Menter D.G., Kopetz S., Hawk E., Sood A.K., Loree J.M., Gresele P., Honn K.V. (2017). Platelet “First Responders” in Wound Response, Cancer, and Metastasis. Cancer Metastasis Rev..

[27] Hou J., Karin M., Sun B. (2021). Targeting cancer-promoting inflammation—Have anti-inflammatory therapies come of age?. Nat. Rev. Clin. Oncol..

[28] Gay L.J., Felding-Habermann B. (2011). Contribution of Platelets to Tumor Metastasis. Nat. Rev. Cancer.

[29] Luo Y., She D.L., Xiong H., Fu S.J., Yang L. (2015). Pretreatment neutrophil to lymphocyte ratio as a prognostic predictor of urologic tumors: A systematic review and meta-analysis. Medicine.

[30] Wei Y., Jiang Y.Z., Qian W.H. (2014). Prognostic Role of NLR in Urinary Cancers: A Meta-Analysis. PLoS ONE.

[31] Huang Y., Gao Y., Wu Y., Lin H. (2020). Prognostic value of systemic immune-inflammation index in patients with urologic cancers: A meta-analysis. Cancer Cell Int..

[32] Yuksel O.H., Verit A., Sahin A., Urkmez A., Uruc F. (2016). White blood cell counts and neutrophil to lymphocyte ratio in the diagnosis of testicular cancer: A simple secondary serum tumor marker. Int. Braz. J. Urol..

[33] Gokcen K., Dundar G., Gulbahar H., Gokce G., Gultekin E. (2018). Can routine peripheral blood counts like neutrophil-to-lymphocyte ratio be beneficial in prediagnosis of testicular cancer and its stages?. J. Res. Med. Sci..

[34] Tan Y.G., Sia J., Huang H.H., Lau W.K.O. (2019). Neutrophil-to-lymphocyte ratio independently predicts advanced pathological staging and poorer survival outcomes in testicular cancer. Investig. Clin. Urol..

[35] Herraiz-Raya L., Moreillo-Vicente L., Martínez-Ruiz J., Agustí-Martínez A., Fernández-Anguita P.J., Esper-Rueda J.A., Salce-Marte L., Armas-Álvarez A., Díaz de Mera-Sánchez Migallón I., Martínez-Alfaro C. (2019). Leukocyte and platelet counts as prognostic values of testicular germ cell tumours. Actas Urol. Esp..

[36] Jankovich M., Jankovichova T., Ondrus D., Breza J. (2017). Neutrophil-to-lymphocyte ratio as a predictor of preoperative tumor staging in testicular germ cell tumors. Bratisl. Lek. Listy.

[37] Fankhauser C.D., Sander S., Roth L., Gross O., Eberli D., Sulser T., Seifert B., Beyer J., Hermanns T. (2018). Systemic inflammatory markers have independent prognostic value in patients with metastatic testicular germ cell tumours undergoing first-line chemotherapy. Br. J. Cancer.

[38] Juan C.A., de la Lastra J.M.P., Plou F.J., Pérez-Lebeña E. (2021). The Chemistry of Reactive Oxygen Species (ROS) Revisited: Outlining Their Role in Biological Macromolecules (DNA, Lipids and Proteins) and Induced Pathologies. Int. J. Mol. Sci..

[39] Baba S.P., Bhatnagar A. (2018). Role Of Thiols in Oxidative Stress. Curr. Opin. Toxicol..

[40] Hanikoglu F., Hanikoglu A., Kucuksayan E., Alisik M., Gocener A.A., Erel O., Baykara M., Cuoghi A., Tomasi A., Ozben T. (2016). Dynamic thiol/disulphide homeostasis before and after radical prostatectomy in patients with prostate cancer. Free. Radic. Res..

[41] Dirican N., Dirican A., Sen O., Aynali A., Atalay S., Bircan H.A., Oztürk O., Erdogan S., Cakir M., Akkaya A. (2016). Thiol/disulfide homeostasis: A prognostic biomarker for patients with advanced non-small cell lung cancer?. Redox Rep..

[42] Bilgin B., Sendur M.A., Hizal M., Kandil S.U., Yaman S., Akıncı M.B., Dede D.Ş., Neselioglu S., Erel Ö., Yalçın B. (2019). Evaluation of dynamic serum thiol-disulphide homeostasis in colorectal cancer. J. Oncol. Sci..

[43] Mukthapura A., Shimogga A., Sudha K.V., Shetty B., Rao G.M. (2010). Oxidative products of proteins and antioxidant potential of thiols in gastric carcinoma patients. J. Med. Biochem..

[44] Suh B., Park S., Shin D.W., Yun J.M., Keam B., Yang H.K., Ahn E., Lee H., Park J.H., Cho B. (2014). Low albumin-to-globulin ratio associated with cancer incidence and mortality in generally healthy adults. Ann. Oncol..

[45] Liu J., Chen S., Geng Q., Liu X., Kong P., Zhan Y., Xu D. (2017). Prognostic value of pretreatment albumin–globulin ratio in predicting long-term mortality in gastric cancer patients who underwent D2 resection. Onco. Targets. Ther..

[46] Tabata F., Wada Y., Kawakami S., Miyaji K. (2021). Serum albumin redox states: More than oxidative stress biomarker. Antioxidants.

[47] Rajab I.M., Hart P.C., Potempa L.A. (2020). How C-Reactive Protein Structural Isoforms With Distinctive Bioactivities Affect Disease Progression. Front. Immunol..

[48] Hart P.C., Rajab I.M., Alebraheem M., Potempa L.A. (2020). C-Reactive Protein and Cancer—Diagnostic and Therapeutic Insights. Front. Immunol..

[49] Bartsch H., Nair J. (2006). Chronic inflammation and oxidative stress in the genesis and perpetuation of cancer: Role of lipid peroxidation, DNA damage, and repair. Langenbeck’s Arch. Surg..

[50] Biswas S.K. (2016). Does the Interdependence between Oxidative Stress and Inflammation Explain the Antioxidant Paradox?. Oxid. Med. Cell. Longev..

[51] Kino K., Hirao-Suzuki M., Morikawa M., Sakaga A., Miyazawa H. (2017). Generation, repair and replication of guanine oxidation products. Genes Environ..

[52] Singh A., Kukreti R., Saso L., Kukreti S. (2019). Oxidative stress: Role and response of short guanine tracts at genomic locations. Int. J. Mol. Sci..

[53] Kino K., Kawada T., Hirao-Suzuki M., Morikawa M., Miyazawa H. (2020). Products of oxidative guanine damage form base pairs with guanine. Int. J. Mol. Sci..

[54] Valavanidis A., Vlachogianni T., Fiotakis C. (2009). 8-Hydroxy-2′ -deoxyguanosine (8-OHdG): A critical biomarker of oxidative stress and carcinogenesis. J. Environ. Sci. Health Part C.

[55] Wiseman H., Halliwell B. (1996). Damage to DNA by reactive oxygen and nitrogen species: Role in inflammatory disease and progression to cancer. Biochem. J..

[56] Shukla S., Srivastava J.K., Shankar E., Kanwal R., Nawab A., Sharma H., Bhaskaran N., Ponsky L.E., Fu P., MacLennan G.T. (2020). Oxidative stress and antioxidant status in high-risk prostate cancer subjects. Diagnostics.

[57] Chuma M., Hige S., Nakanishi M., Ogawa K., Natsuizaka M., Yamamoto Y., Asaka M. (2008). 8-Hydroxy-2′-deoxy-guanosine is a risk factor for development of hepatocellular carcinoma in patients with chronic hepatitis C virus infection. J. Gastroenterol. Hepatol..

[58] Chang D., Wang F., Zhao Y.S., Pan H.Z. (2008). Evaluation of oxidative stress in colorectal cancer patients. Biomed. Environ. Sci..

[59] Bahar G., Feinmesser R., Shpitzer T., Popovtzer A., Nagler R.M. (2007). Salivary analysis in oral cancer patients: DNA and protein oxidation, reactive nitrogen species, and antioxidant profile. Cancer.

[60] Kubo N., Morita M., Nakashima Y., Kitao H., Egashira A., Saeki H., Oki E., Kakeji Y., Oda Y., Maehara Y. (2014). Oxidative DNA damage in human esophageal cancer: Clinicopathological analysis of 8-hydroxydeoxyguanosine and its repair enzyme. Dis. Esophagus.

[61] Ma Y., Zhang L., Rong S., Qu H., Zhang Y., Chang D., Pan H., Wang W. (2013). Relation between gastric cancer and protein oxidation, DNA damage, and lipid peroxidation. Oxid. Med. Cell. Longev..

[62] Kumar A., Pant M.C., Singh H.S., Khandelwal S. (2012). Determinants of oxidative stress and DNA damage (8-OhdG) in squamous cell carcinoma of head and neck. Indian J. Cancer.

[63] Qing X., Shi D., Lv X., Wang B., Chen S., Shao Z. (2019). Prognostic significance of 8-hydroxy-2′-deoxyguanosine in solid tumors: A meta-analysis. BMC Cancer.

